# *In Vivo* Quantitative Monitoring of Drug Release from Halo-Spun Rubbery Mats by Fluorescent Organism Bioimaging (FOBI)

**DOI:** 10.3390/polym17222972

**Published:** 2025-11-07

**Authors:** Peter Polyak, Aswathy Sasidharan Pillai, Laszlo Forgach, Kristof Molnar, Judit E. Puskas, Domokos Mathe

**Affiliations:** 1 Department of Food, Agricultural and Biological Engineering, College of Food, Agricultural, and Environmental Sciences, The Ohio State University, 1680 Madison Avenue, Wooster, OH 44691, USA; polyak.10@osu.edu (P.P.); aswathygeetha4@gmail.com (A.S.P.); molnar.kristof1@semmelweis.hu (K.M.); puskas.19@osu.edu (J.E.P.); 2 Laboratory of Nanochemistry, Department of Biophysics and Radiation Biology, Semmelweis University, Nagyvarad ter 4, 1094 Budapest, Hungary; laszlo.forgach@gmail.com; 3 Hungarian Center of Excellence for Molecular Medicine (HCEMM), In Vivo Imaging Advanced Core Facility, Semmelweis University Site, Tuzolto u. 37-47, 1094 Budapest, Hungary; 4 Department of Biophysics and Radiation Biology, Semmelweis University, HUN-REN TKI, Tűzoltó u. 37-47, 1094 Budapest, Hungary

**Keywords:** *in vivo* drug release, Fluorescent Organism Bioimaging FOBI, Doxorubicin.HCl, drug release model, local cancer chemotherapy

## Abstract

This paper will present *in vivo* release profiles of Doxorubicin.HCl from halo-spun drug-loaded rubbery porous mats. For the very first time, Fluorescent Organism Bioimaging (FOBI) was used to follow drug release in a live animal model with induced tumors. A new predictive model based on apparent diffusion coefficients to simulate release profiles will also be presented and could have general applications for release profile predictions. Surprisingly, histological evaluation found that the tissue layer forming next to the drug-eluting mats had unordered morphology and only necrotic cells. This is a stunning contrast to the highly regular collagen structure next to mats without the drug, typical of an adverse foreign body type reaction. The findings suggest that this drug-eluting fiber mat can be used as a local chemotherapy approach coupled with mitigation of capsular contracture, the major complication associated with breast reconstruction following mastectomy.

## 1. Introduction

In 2022, in the United States alone, 1,851,238 new cancer cases were reported, and 613,349 people died of the disease. The most frequently diagnosed cancer in women was breast cancer, with 287,850 new cases diagnosed in 2022, and over 40,000 deaths [1]. In current treatment models, mastectomy with postoperative chemotherapy offers the best rate of survival. Of those patients, over 50% opt for breast reconstruction. Current tissue expanders and implant materials for reconstruction are silicone rubber-based and have over 20% complication rates, including implant hardening, tissue necrosis, gel bleed, and capsular contracture [2,3,4]. In capsular contracture, a thick, fibrous capsule forms around most implants, leading to pain, disfigurement and possible rupture, often necessitating further surgeries. A total of 35–50% of reconstruction patients require revisional surgery within 6 years [5]. Furthermore, up to 80% of silicone breast implants are removed or replaced within 8–10 years. This is a major problem considering over 300,000 American women received silicone rubber breast implants in 2020 alone [5]. Thus far, physical and chemical modifications have been attempted in order to solve the problem of capsular contracture, including slow local drug release [6,7,8].

In addition to mitigating capsular contracture, localized chemotherapy seems to be an interesting new approach to breast cancer treatment. The concept of local chemotherapy could offer reduced side effects as compared to those associated with systemic chemotherapy. Local intra-tumor delivery of a Doxorubicin (DOX)-labeled polymeric drug conjugate has already been shown to be more effective than systemic intravenous delivery in a hepatocellular carcinoma model [9]. This approach may also prevent recurrence; sadly, 19% of post-mastectomy patients are also subject to recurrent cancer (2–3 years average).

Our research group has been investigating drug encapsulation into polymer films and fiber mats with sustained release, focusing on polyisobutylene (PIB)-based thermoplastic elastomers (TPEs) because of the excellent bio-inertness and biostability of PIB. A linear poly(styrene-*b*-isobutylene-*b*-styrene) triblock TPE (L_SIBS) is used in clinical practice as the drug-eluting coating on the TAXUS^TM^ coronary stent [10]. Over 10 million patients have benefited from this device, emphasizing the significance of PIB-based biomaterials. Due to the unique low permeability of PIB-based materials such as L_SIBS, sustained drug delivery is achieved, but reportedly only approximately 10% of the encapsulated drug, Taxol, elutes from the coating, which is therapeutic for this application [10].

With the expiration of patent protection for L_SIBS, we have developed new generations for drug-delivery applications [11,12,13,14]. The latest PIB-based TPE our group developed is poly(alloocimene-*b*-isobutylene-*b*-alloocimene) (AIBA) [11,12,13]. This TPE is much easier to produce than L_SIBS and we can produce kg quantities in the laboratory. We have recently shown that electrospun AIBA mats loaded with the anti-inflammatory drug Zafirlukast (ZAF) locally released *in vivo* in rats lowered inflammation and fibrous capsule development compared to oral ZAF treatment [8].

This paper will present a new method for *in vivo* monitoring of local DOX.HCl release from drug-loaded AIBA-based mats produced with a patented halo-spinning method [15] with Fluorescent Organism Bioimaging (FOBI), compare *in vitro* and *in vivo* release profiles, and present a new predictive model to simulate release profiles. The results constitute a step towards proving the hypothesis for the superiority of local sustained cancer drug delivery.

## 2. Materials and Methods

### 2.1. Materials

Poly(alloocimene-*b*-isobutylene-*b*-alloocimene) (AIBA) was synthesized as reported in our previous paper [13]. Poly(ethylene glycol) (PEG) (M_n_ = 2000 g/mol) was purchased from Sigma-Aldrich (USA) and was used as received. Doxorubicin hydrochloride (DOX.HCl, CAS 25316-40-9) was purchased from AvaChem Scientific (San Antonio, TX, USA). Phosphate-buffered saline (PBS) powder (pH 7.4) packed in foil pouches was purchased from Sigma-Aldrich (Burlington, MA, USA), and 0.01 M PBS solution was freshly prepared by dissolving each pouch in one liter distilled water.

### 2.2. Preparation of DOX.HCl-Loaded Fiber Mats

Fiber mats were prepared from a solution of DOX.HCl/AIBA/PEG by a proprietary halo-spinning process by Gelatex [15] (Tallinn, Estonia). The patented method utilizes compressed air and the centrifugal force exerted by revolving spinnerets to form fibers and direct them toward the carrier layer that collects the fibers [15]. As a carrier layer, poly(tetrafluoro-ethylene) (Teflon, AmericanDurafilm, Holliston, MA, USA) was used.

### 2.3. Scanning Electron Microscopy (SEM)

SEM measurements were performed using a Hitachi Schottky Field Emission SU5000 instrument (Hitachi, Santa Clara, CA, USA). Micrographs were recorded after sputter coating the samples with gold at ambient temperature. The accelerating voltage was 5 kV; the imaging was based on the detection of secondary electrons. The magnification factor varied between 100 and 500.

### 2.4. X-Ray Photoelectron Spectroscopy (XPS)

XPS spectra were collected using a Thermo Fisher Nexsa G2 XPS (Thermo Fisher Scientific, Waltham, MA, USA) System utilizing an Al Kα source (15 kV, 60 W). All the spectra were calibrated with reference to the aliphatic C—C peak binding energy at 284.8 eV. The curves were fitted using the CasaXPS 2.3.25 software with a combined Gaussian-Lorentzian profile after subtracting the background.

### 2.5. In Vitro Release Studies

DOX.HCl release studies were performed using the DOX.HCl-loaded AIBA/PEG mats in phosphate-buffered saline (PBS) solution at 37 °C. 1 cm × 1 cm square samples were used for the studies in triplicates. Because the UV signal intensities were too low for the 5 wt% samples in 100 mL PBS, the release profiles were measured in 10 mL PBS. Table 1 lists the samples.

The samples were placed into stainless-steel mesh sample holders, which were immersed in beakers containing 100 or 10 mL 0.01 M PBS solution (pH 7.4), freshly prepared before each experiment. The beakers were placed in a water bath thermostated at 37 °C, magnetic stirrer set to 200 RPM. The beakers were covered with parafilm to avoid evaporation of the PBS, and the entire experimental setup was covered with aluminum foil to protect the samples from light. At specified time intervals (5, 10, 15, 20, 30, 40, 50, 60, 75, 90, 105, 120, 180, 270, and 1500 min), the liquid in the beakers was removed and refilled with 10 mL freshly prepared PBS solution pre-heated to 37 °C. 2 mL of the 100 mL removed liquid was subjected to UV-VIS analysis using a Molecular Devices SpectraMax Plus 384 spectrophotometer. Absorbance values were converted to DOX.HCl concentrations by using a calibration curve that was obtained as follows: DOX.HCl solutions were prepared in PBS buffer (pH 7.4) with varying concentrations (0.2, 0.5, 1.0, 1.5, and 2.0 mg/L). Then, the absorbance of the solutions was measured using the UV-VIS spectrophotometer in the 200–600 nm wavelength range. Absorbance values recorded at 480 nm were plotted against the concentration values; the equation of the regression line fitted onto these points was used to convert absorbances to concentrations (see Figure S1).

### 2.6. Animal Preparation and Surgery

NMRI FOXn1 nu/nu mice from Janvier (Bretagne, France) were utilized in this study. The “short term” group consisted of *n* = 1 control mouse having an unloaded fiber mat and *n* = 5 mice with DOX.HCl-loaded fiber mats for 30 days. Another control group of *n* = 2 mice with no tumors were implanted with unloaded fiber mats for a period of three months. The animals were provided *ad libitum* access to food and water and were housed under controlled conditions for temperature, humidity, and light. All procedures adhered to ARRIVE guidelines and complied with the European Communities Council Directive (86/609 EEC), approved by the Animal Care and Use Committee of Semmelweis University (protocol number: PE/EA/293-7/2021). The mice were 30–37 weeks old with an average body weight of 34.1 ± 7.6 g.

Ten weeks before the study he left hind legs of the 6 mice in the short-term study was subcutaneously transplanted with a small volume of breast cancer cell line expressing endothelin receptor and HER-2 antigen (ZR-71-1) cells, generously provided by the Department of Immunology at Carl Gustav Carus University (Dresden, Germany). Tumor volume was monitored using three-dimensional magnetic resonance imaging. The body weight and general well-being of the mice were assessed weekly.

The implantation surgical procedure was conducted under isoflurane anesthesia (5% for induction and 1.5–2% for maintenance; Arrane^®^, Baxter, Newbury, UK). An incision was made with surgical scissors on the left posterior caudal side of the tumors, and a hole was prepared using blunt forceps. Circular samples with a diameter of 4.5 mm were inserted into the hole, and the subcutaneous incision was closed using Radik-PGLA 4-0 filament (Kollsut International Inc., Miami, FL, USA). Stitches were removed after 10 days.

### 2.7. Fluorescent Organism Bioimaging (FOBI)

The fluorescence of DOX.HCl-loaded and control specimens was imaged using a two-dimensional epifluorescent optical imaging instrument (Fluorescent Organism Bioimaging Instrument, Neoscience Co., Ltd., Suwon-si, Korea). Mice were anesthetized with isoflurane (5% for induction and 1.5–2% to maintain the appropriate level of anesthesia; Baxter, AErrane). The images were collected after shaving to remove hair at different time points (pre- and post-surgery, 4 h; 1, 2, 3, 9, 15, 22 and 29 days post-surgery) with an excitation wavelength of 480 nm corresponding to the excitation maximum of DOX.HCl (ex: 494 nm; em: 512 nm in water). The emission spectrum of DOX.HCl was in the pass band of the used emission filter. Image acquisition parameters were the following: exposure time: 2000 ms and gain: 1. The images were evaluated with VivoQuant 5.4.3 software (Invicro-Konica Minolta, Boston, MA, USA). The amount of released DOX.HCl was calculated from the decrease in the DOX.HCl in the mats.

Off-line calibration was carried out as follows: 4.8 mg DOX.HCl was dissolved in 2 mL PBS buffer contained in an Eppendorf tube. Then, a sequence of solutions with decreasing concentrations was created. Each sample in the sequence was analyzed with FOBI. Data points were used to establish the region where linear correlation was found between fluorescent intensity and DOX.HCl concentration. The values were shifted by the background fluorescence intensity of the Eppendorf tube (~15 units). The calibration plot is shown in Figure S2A.

### 2.8. Histology

For the histological evaluation, samples were obtained from all experimental groups from all animals. Tissue samples including the tissue pockets and the implanted mats were prepared and sectioned using a Leica CM1950 microtome (Leica Biosystems Nussloch GmbH, Nussloch, Germany), perpendicular to the surface of the implant. The 7 μm thick sections were stained with hematoxylin and eosin (HE) in the routine manner [16]. The slides were scanned with a Pannoramic Midi II slide scanner instrument (3DHistech, Budapest, Hungary), the digital slides were analyzed, and the representative pictures were taken with the SlideViewer software (3DHistech, Budapest, Hungary).

## 3. Results and Discussion

### 3.1. Characterization of the Halo-Spun DOX.HCl-Loaded Samples

**Figure 1:**
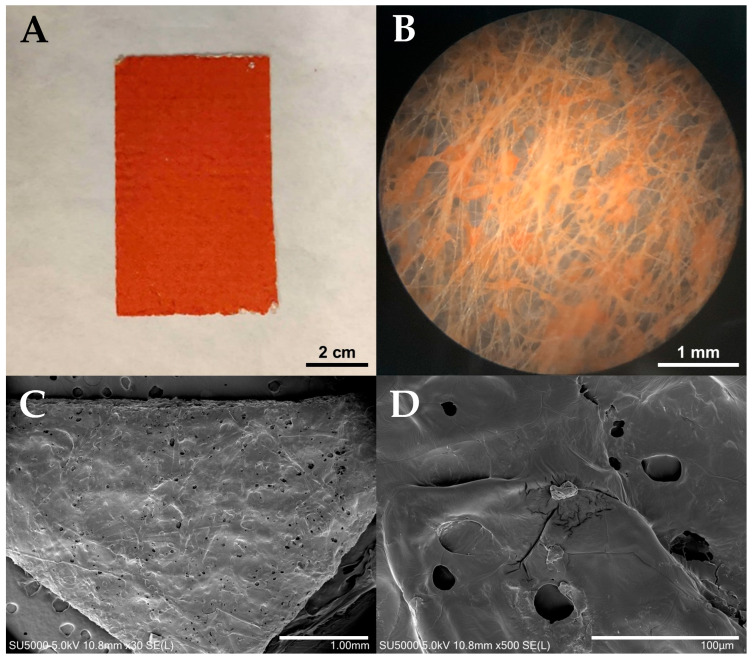
Macroscopic appearance (**A**), optical (**B**) and SEM micrographs (**C**,**D**) of the mats.

The DOX.HCl-loaded samples had intense orange color, as shown in Figure 1A. Optical microscopy showed the distribution of DOX.HCl in the mat (Figure 1B). Halo-spinning produced a porous mat, as shown in the SEM images of a representative halo-spun sample (Figure 1C,D).

XPS did not detect any DOX.HCl or PEG on the surface. The unique property of PIB-based thermoplastic elastomers such as AIBA is that even when spun from a solution of the polymer/drug mixtures the surface is covered with a ~10 nm layer of pure PIB on account of the low surface energy, resulting in complete incorporation of DOX.HCl into the polymer. DOX.HCl was selected for *in vivo* monitoring because it fluoresces in the red and can be readily distinguished from the green fluorescence of live tissues [9]. First, *in vitro* release studies were conducted.

### 3.2. In Vitro Release Studies

#### 3.2.1. Calibration

To quantify the amount of DOX.HCl released in PBS first a calibration plot needed to be created. In the UV-Vis spectrum of DOX.HCl absorptions at 260 and 480 nm (the two 1A-1La and 1A-1Lb π-π* transitions polarized along the short and long axis of the molecule) were identified, with a very small signal between 350 and 370 nm assigned to the three carbonyl groups in the DOX.HCl molecule [17]. The absorption at 480 nm was used for calibration and to quantify DOX.HCl release from the samples (see Figure S1A,B).

#### 3.2.2. Investigation of *In Vitro* DOX.HCl Release

**Figure 2:**
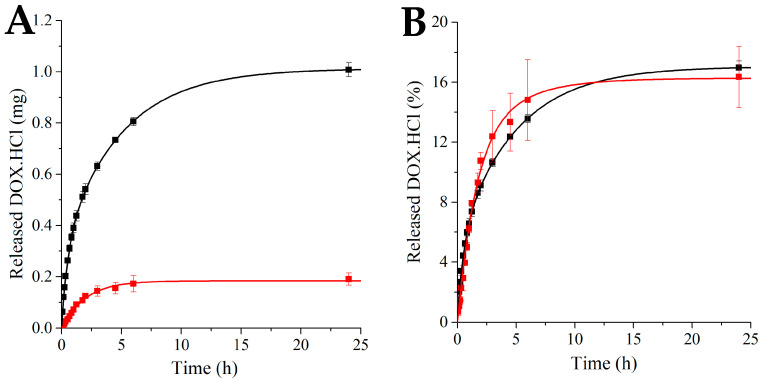
Time dependence of *in vitro* DOX.HCl release; the measurements were repeated three times. ■: 20 wt% DOX.HCl; ■: 5 wt% DOX.HCl.

Samples of size 1 cm^2^ with 5 and 20 wt% DOX.HCl loading were tested in triplicates. The time dependence of DOX.HCl release is shown in Figure 2. The drug is released rapidly at the beginning of the experiment. However, the rate of the release decreased after about 3 h and leveled off at about 6 h for the 5 wt% sample. Literature search revealed that DOX.HCl is unstable in phosphate-buffered solution at 37 °C, with a half-life of 50 h [18,19]. Interestingly, the % release profile is very close for the two loadings.

The initial region (up to 5% release) was plotted using Peppas’ equation [20]:

(1) $$\frac{M_{t}}{M_{\infty }}=k\cdot t^{n}$$

According to Peppas, *n* = 0.5 for thin films indicates Fickian diffusion, 0.5 < *n* < 1 characterizes anomalous non-Fickian diffusion, while *n* = 1 indicates a zero-order profile. For the 20% DOX.HCl content *n* = 0.793 with R^2^ = 0.9953 was found. This value indicates that DOX.HCl was released by anomalous (non-Fickian) transport. The 5 wt% sample yielded *n* = 0.483 with R^2^ = 0.9965 indicating Fickian diffusion up to ~5% release. However, in the later stages, the process deviates from the Fickian mechanism. It should be noted that electrospun fiber mats loaded with Zafirlukast displayed Fickian diffusion up to 60% release [14].

Based on the *in vitro* data and preliminary *in vivo* test results showing necrosis with the 20 wt% loading, samples with 5 wt% DOX·HCl loading were implanted into 5 mice to demonstrate the feasibility of monitoring DOX·HCl release *in vivo* using FOBI.

### 3.3. Development of the New In Vivo Monitoring Method

**Figure 3:**
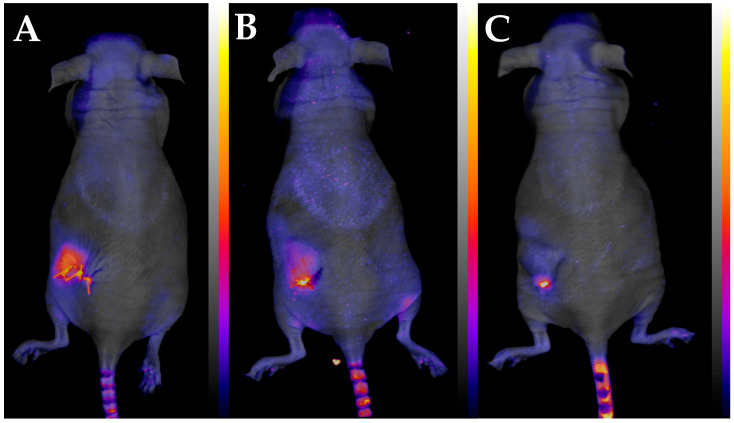
Images taken of a mouse right after implantation (**A**), after 2 days (**B**), and after 22 days (**C**).

FOBI has been used for live cell monitoring [21,22,23], but according to our knowledge *in vivo* drug release monitoring has never been reported. We decided to try this approach using six mice. One mouse was implanted with an unloaded sample while 5 mice had samples loaded with 5 wt% DOX.HCl. Based on the *in vitro* samples, the 4.5 mm disks contained 0.1845 mg DOX.HCl. Figure 3 shows representative images taken right after implantation, after 2 days and the end of the *in vivo* monitoring. As the drug leaves the implant, its fluorescent intensity decreases.

The off-line calibration (see Experimental and Figure S2A) demonstrated the unique sensitivity and linear correlation of the monitoring in the 0–1.5 µg concentration range. Fluorescent intensity values in the porous mats measured *in vivo* are listed in Table S1. The background intensity measured on the animal with the control mat with no DOX.HCl were deducted from the intensity values measured on the animals implanted with mats containing DOX.HCl. The values were then converted to absolute DOX.HCl weights by using a linear correlation with the nominal initial DOX.HCl amount (0.1845 mg) as the maximum value. The linear calibration plot obtained this way is displayed in Figure S2B. The amount of released DOX.HCl was calculated from the decrease measured in the mats by FOBI.

**Figure 4:**
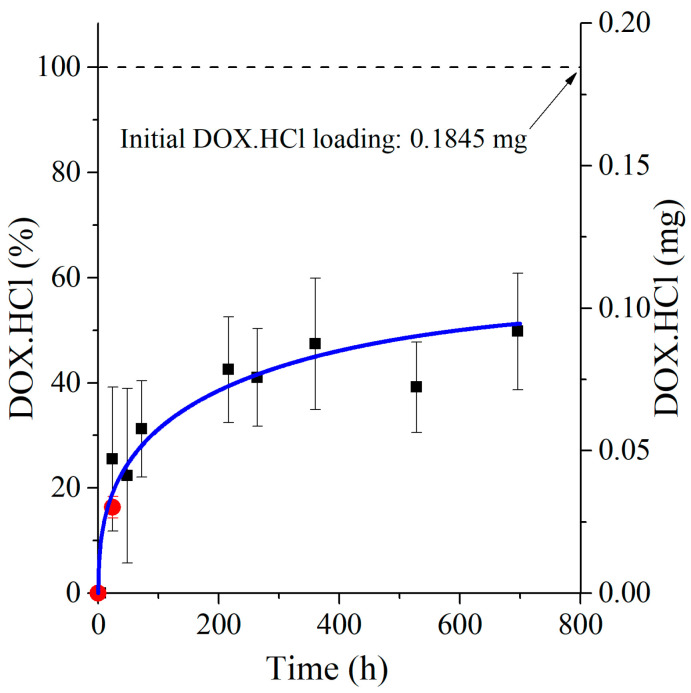
Percentage and amount of released DOX.HCl plotted against time. ■: *In vivo* release; ●: *In vitro* release; solid blue line: calculated data.

The released amounts from the data averaged from five animals are shown in Figure 4 (black squares). While the error bars are large, a clear release profile emerges. Importantly, the 24 h release % is in good agreement with the *in vitro* data.

Figure 4 also displays the simulated release profile based on a computational method using the concept of apparent diffusion coefficients changing with the spatial coordinates of the drug-loaded mat—see Section 3.4. The agreement is excellent.

### 3.4. Mathematical Model Development and Comparison with Measured Data

Sirianni et al. analyzed release of Taxol from stents coated with L_SIBS at 8.8, 25 and 35 wt% loading [24]. They considered Fickian diffusion, surface dissolution, bulk dissolution and osmotic gradient models. Their analysis concluded that the Fickian model combined with surface dissolution described the release profiles at all loading levels, but especially at 25 and 35 wt% loading where initial burst release was observed. At the 8.8 wt% therapeutic loading used in the stent coatings, 10% elution was reached in 330 days.

**Figure 5:**
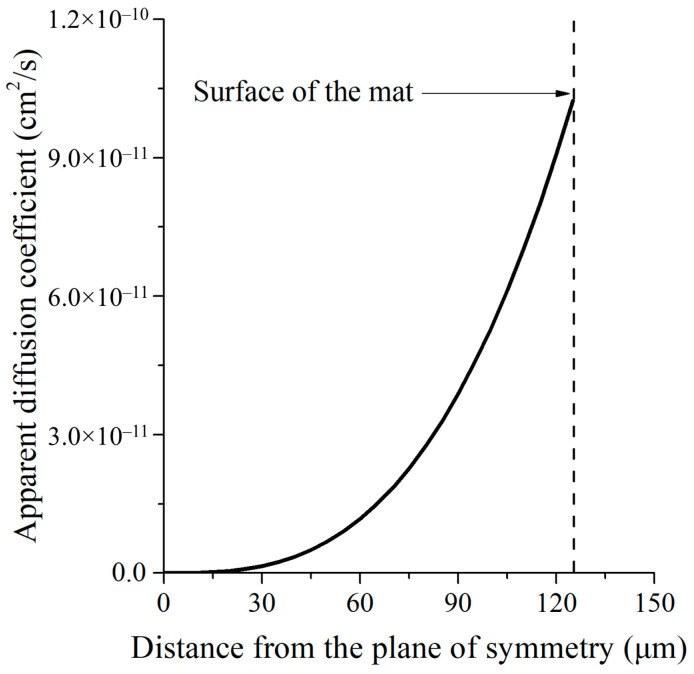
Spatial coordinate dependence of the apparent diffusion coefficient.

Our new model proposes the use of a computational method based on the concept of apparent diffusion coefficients. In this approach, the inhomogeneities (pores, etc.) that affect the rate of molecular transport are considered by introducing apparent diffusion coefficients, changing with the spatial coordinate.

(2) $$\frac{\partial c}{\partial t}=D_{a}\left(x\right)\frac{\partial^{2}c}{\partial x^{2}}+\frac{dD}{dx}\frac{\partial c}{\partial x}$$

where *c* is the concentration of the drug, *x* and *t* refer to the spatial coordinate and time, respectively, and *D_a_* is the apparent diffusion coefficient that depends on the spatial coordinate. This dependence is approximated with the function in Equation (3):

(3) $$D_{a}\left(x\right)=a\cdot x^{b}$$

where *a* and *b* are adjustable fitting parameters. The solution to Equation (2) was found numerically using purpose-specific software developed by our research team in MATLAB (R2025b Version 25.2.0.3042426). The *c*(*x*,*t*) surface function is presented in Figure S3 and JEP_DOX_Video. and the *a* and *b* parameters of *D_a_*(*x*) (Equation (3)) were optimized using an additional software written in MATLAB that iteratively found the best fit to experimental data. Best fit was achieved using *a* = 4.722 × 10^−8^ and *b* = 2.948. Figure 5 shows the dependence of the diffusion coefficient on the distance from the plane of symmetry.

Forrey calculated the diffusion coefficient of tetracycline (TAC) in SIBS and reported it to fall in the *D* ~ 10^−11^ cm^2^/s region [25] in good agreement with the ranges in Figure 5. Thus, the apparent diffusion coefficient is largest at the surface and decreases towards the inner parts of the sample.

Although extrapolation beyond measured data ranges may be misleading, the time required for 100% release was estimated to be 1.8–2 years. Note that this value is a result of extrapolation and must be handled with caution. However, as an estimation, it highlights slow sustained release from the porous implant, in comparison with the 10% Taxol released from the Taxus coronary stent in one year [26,27].

### 3.5. Histology

**Figure 6:**
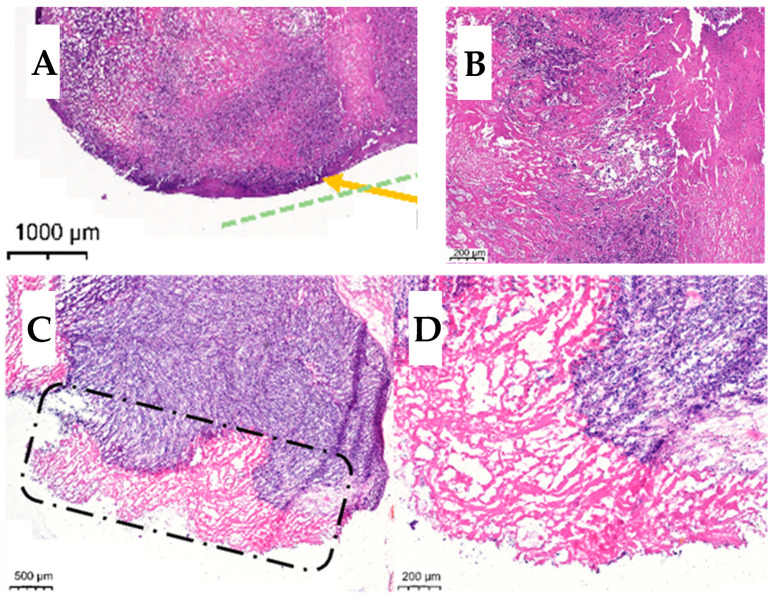
Histology of tumor tissue next to unloaded (**A**,**B**) and DOX.HCl-loaded (**C**,**D**) implants. The surface of the collagen-rich capsule is marked by a yellow arrow in Panel (**A**) (see the region above the green dashed line). The preventive chemotherapeutic barrier is highlighted by a dash-dot marker in Panel (**C**) and magnified in Panel (**D**).

While the major goal of this work was to monitor drug release *in vitro* using FOBI, unexpected discoveries have also been made from the histology data. Comparison of the histology of control mats and DOX.HCl-eluting mats revealed that the latter had a major influence on foreign body type reaction. The control mats had the collagen-rich capsule usually forming due to an inflammatory reaction next to any foreign body *in vivo.* The thickness of the capsules was measured to be 70.8 ± 39.9 µm on the 30-day control implant and 82.3 ± 62.5 µm on the 90-day control implants—see Figure 6A,B panels. Live cancer cells (blue stain) are present in the tumor, which could lead to metastasis. In contrast, panels C and D in Figure 6 demonstrate that the drug-eluting implants had a layer of necrotic tissue (pink stain) in between the tumor and the implant, forming a “preventive chemotherapeutic barrier” against tumor metastasis.

In control animals (both 30 days and 3 months), cell division and outward growth caused central hypoxia, central necrosis and a large peripheric rim of viable tumor cells, responsible for further tumor growth and metastasis. In contrast, the release of DOX.HCl from the mats clearly caused a peripheric necrotic effect in the tumors, thereby forming a “preventive chemotherapeutic barrier” that could prevent tumor metastasis.

Additionally, the histological data showed that the “preventive chemotherapeutic layer” forming in between tumors and drug-eluting implants had disordered morphology with only necrotic cells, as opposed to the highly regular collagen structure on the implants without the drug.

## 4. Conclusions

This work successfully demonstrated live animal monitoring of DOX.HCl release from rubbery fiber mats using FOBI for the first time. The data was compared with *in vitro* drug release data, and a mathematical model was developed using the concept of apparent diffusion coefficients. The model yielded a profile in good agreement with experimental data. Additionally, histological data showed that a “preventive chemotherapeutic layer” formed in between tumors and drug-eluting implants and had disordered morphology with only necrotic cells, as opposed to the highly regular collagen structure on the implants without the drug. Mass production of the drug-eluting mat is possible using the patented halo-spinning technique that produced drug-loaded fiber mats on a continuous line, demonstrating the scalability of production [15].

## Figures and Tables

**Table 1:** Samples for *in vitro* testing.

Nominal DOX.HCl Load (wt%)	Sample Mass (mg)	Average Mass (mg)	Nominal DOX.HCl Content (mg)
20 wt%	32.0 26.8 30.3	29.7	5.94
5 wt%	24.5 23.0 22.4	23.3	1.17

## Data Availability

Data including images and readout data files, are available upon reasonable request from the corresponding author.

## Supplementary Materials

The following supporting information can be downloaded at: https://www.mdpi.com/article/10.3390/polym17222972/s1, DOX_SI.docx: Figure S1: UV-Vis absorption spectra of DOX.HCl in PBS buffer (A) and the regression line fitted to the absorbances measured at 480 nm (B); Figure S2: Results of the offline calibration without the background signal (A), and the DOX.HCl amount—fluorescent intensity correlation plot (B); Figure S3: Particular solution of Equation (2), i.e., the c(x,t) surface function; Table S1: Fluorescent intensity values in the porous mats measured *in vivo*; JEP_DOX_Video.mp4.
