# Supplementary material for: In Vivo Quantitative Monitoring of Drug Release from Halo-Spun Rubbery Mats by Fluorescent Organism Bioimaging (FOBI)
Source: Polymers (Basel). 2025 Nov 7;17(22):2972. doi: 10.3390/polym17222972 (PMC12656612; doi:10.3390/polym17222972)
Supplement: Supplementary file 1 [file polymers-17-02972-s001.zip › JEP_2025_10_17_DOX_SI.pdf]

In Vivo Quantitative Monitoring of Drug Release from Halo-Spun Rubbery Mats by Fluorescent Organism Bioimaging (FOBI)

Supplementary Information

Peter Polyak[1], Aswathy Sasidharan Pillai[1], Laszlo Forgach[2,3], Kristof Molnar[1,2], Judit E. Puskas[1], Domokos Mathe[3,4*]

[1]Department of Food, Agricultural and Biological Engineering, College of Food, Agricultural, and Environmental Sciences, The Ohio State University, 1680 Madison Avenue, Wooster, OH 44691, USA

[2]Laboratory of Nanochemistry, Department of Biophysics and Radiation Biology, Semmelweis University, Nagyvarad ter 4, 1094 Budapest, Hungary

[3]Hungarian Center of Excellence for Molecular Medicine (HCEMM), In Vivo Imaging Advanced Core Facility, Semmelweis University Site, Tuzolto u. 37-47, 1094 Budapest, Hungary

[4]Department of Biophysics and Radiation Biology, Semmelweis University, HUN-REN TKI, Tűzoltó u. 37-47, 1094 Budapest, Hungary

*Corresponding author: Domokos Mathe, E-mail: mathe.domokos@semmelweis.hu

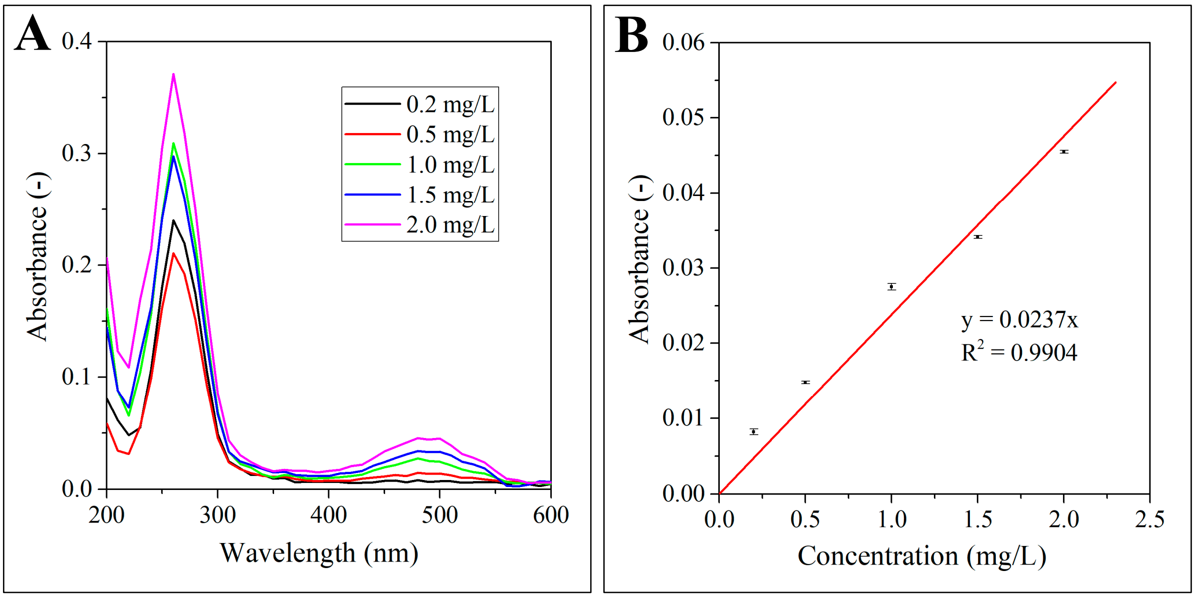

Figure S1: UV-Vis absorption spectra of DOX.HCl in PBS buffer (**A**) and the regression line fitted to the absorbances measured at 480 nm (**B**).

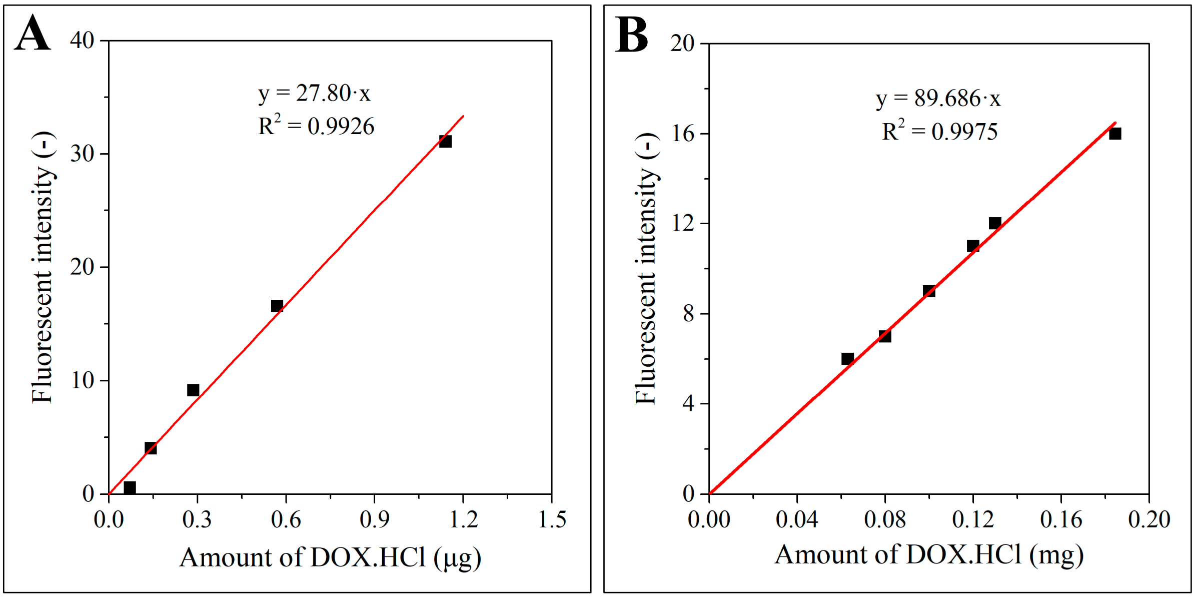

Figure S2: Results of the offline calibration without the background signal (**A**), and the DOX.HCl amount – fluorescent intensity correlation plot (**B**).

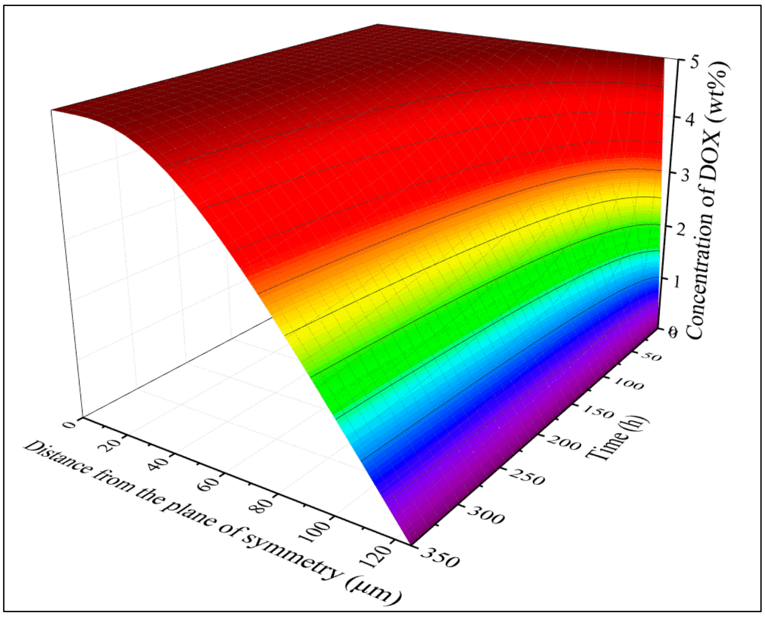

Figure S3: Particular solution of **Equation (2)**, i.e., the c(x,t) surface function

<u>Boundary conditions</u>

A particular solution of **Eq. 2** was found numerically by using static boundary conditions. The concentration of DOX.HCl outside the investigated mat is approximated to equal zero at all times. This boundary condition is often referred to as 'infinite dilution', i.e., the concentration of DOX.HCl in the dissolution medium (in the case of *in vivo* measurements, the bodies of the mice) is so small that approximating it with zero does not bias the result significantly. Graphically, this boundary condition is represented by the right edge of the surface shown in **Fig. SI3**, which converges to zero at all time coordinates.

<u>Initial conditions</u>

The initial conditions for the differential equation provided as **Eq. 2** are determined by the concentration profile of DOX.HCl at the t = 0 h time coordinate (i.e., at the beginning of the experiment). At this time coordinate, the concentration of DOX.HCl equals 5 wt% across the entire width of the mat. As discussed above, the concentration equals zero outside the mat.

The initial conditions are represented graphically by the concentration profile at t =0 h, i.e., the edge of the *c(x,t)* surface at the t =0 h time coordinate. The t =0 h concentration profile consists of points equaling 5 wt% inside the mat and 0 wt% outside the mat.

Avoiding redundancy

As DOX.HCl leaves the carrier on both sides of the mat, simulating the dissolution across the entire 250 μm width would lead to redundancy. In order to avoid handling the problem redundantly, the release of the drug was simulated in one half of the mat. The mats can be considered homogeneous; therefore, the dissolution proceeds in both halves of the mat with the same kinetics. Accordingly, **Eq. 2** was numerically solved in one half of the mat (hence the 250/2=125 μm-wide spatial coordinate axis showing the distance from the plane of symmetry). Lastly, the calculated amount of DOX.HCl that left the mat must be multiplied by two to obtain the accurate final result.

Table S1: Fluorescent intensity values in the porous mats measured *in vivo*.

Time (h)	Mouse 01	Mouse 02*	Mouse 03	Mouse 04	Mouse 05	Mouse 06 (control)	Average Intensity	Intensity with no background
pre-surgery (0)	24	20	28	19	25	20	23	0
4	30	49	29	34	52	21	39	16
24	29	34	29	34	44	21	34	11
48	29	33	29	37	47	25	35	12
72	29	34	27	36	36	23	32	9
216	30	26	27	32	36	26	30	7
264	28	28	28	33	37	26	30	7
360	28	23	36	28	32	24	29	6
528	32	29	34	26	35	22	31	8
696	31	25	34	30	22	21	28	5
**Average background**						**23**		

*Average of 2 measurements
